# Urinary Acrylonitrile Metabolite Concentrations Before and after Smoked, Vaporized, and Oral Cannabis in Frequent and Occasional Cannabis Users

**DOI:** 10.3390/ijerph17186438

**Published:** 2020-09-04

**Authors:** David L. Ashley, Víctor R. De Jesús, Osama A. Abulseoud, Marilyn A. Huestis, Daniel F. Milan, Benjamin C. Blount

**Affiliations:** 1 Department of Population Health Sciences, School of Public Health, Georgia State University, Atlanta, GA 30303, USA; 2 Tobacco and Volatiles Branch, Division of Laboratory Sciences, National Center for Environmental Health, Centers for Disease Control and Prevention, Atlanta, GA 30341, USA; foa5@cdc.gov (V.R.D.J.); bkb3@cdc.gov (B.C.B.); 3 Chemistry and Drug Metabolism Section, Intramural Research Program, National Institute on Drug Abuse, National Institutes of Health, Baltimore, MD 21224, USA; abulseoud@gmail.com; 4 Institute for Emerging Health Professions, Thomas Jefferson University, Philadelphia, PA 19107, USA; marilyn.huestis@gmail.com; 5 Robert J. Tomsich Pathology & Laboratory Medicine Institute, Cleveland Clinic Foundation, Cleveland, OH 44195, USA; daniel.f.milan7@gmail.com

**Keywords:** acrylonitrile, urine exposure biomarker, cannabis, exposure routes, half-life

## Abstract

Cannabis use through smoking, vaping, or ingestion is increasing, but only limited studies have investigated the resulting exposure to harmful chemicals. N-acetyl-S-(2-cyanoethyl)-L-cysteine (2CYEMA), a urinary metabolite of acrylonitrile, a possible carcinogen, is elevated in the urine of past-30-day cannabis users compared to non-cannabis users. Five frequent and five occasional cannabis users smoked and vaped cannabis on separate days; one also consumed cannabis orally. Urine samples were collected before and up to 72 h post dose and urinary 2CYEMA was quantified. We compared 2CYEMA pre-exposure levels, maximum concentration, time at maximum concentration for occasional versus frequent users following different exposure routes, and measured half-life of elimination. Smoking cannabis joints rapidly (within 10 min) increased 2CYEMA in the urine of occasional cannabis users, but not in frequent users. Urine 2CYEMA did not consistently increase following vaping or ingestion in either study group. Cigarette smokers had high pre-exposure concentrations of 2CYEMA. Following cannabis smoking, the half-lives of 2CYEMA ranged from 2.5 to 9.0 h. 2CYEMA is an effective biomarker of cannabis smoke exposure, including smoke from a single cannabis joint, however, not from vaping or when consumed orally. When using 2CYEMA to evaluate exposure in cannabis users, investigators should collect the details about tobacco smoking, route of consumption, and time since last use as possible covariates.

## 1. Introduction

Although federal law does not allow its use, more and more states are legalizing the retail non-medical adult use of cannabis (most commonly known as marijuana) [1,2]. Cannabis use peaked in the late 1970s, declined until the early 1990s, but has been rising over the last 30 years [3]. Between 2006 and 2017, the annual prevalence of cannabis use in the 19–28 age group increased from 27.7% to 37.5% and for those age 35, from 11.4% to 23.8%. Daily marijuana use in the 19–28 age group increased from 5.0% in 2006 to 7.8% in 2017 and for those age 35 from 1.9% in 2006 to 5.1% in 2017 [3]. Even though edibles and vaping are substantial routes of use by adolescents, smoking is still the most frequent means of consuming cannabis [4,5,6]. With legalization, adults and adolescents increasingly view cannabis consumption as harmless [7,8]. As use increases, public health issues such as driving while intoxicated, fatal car crashes [9], unintentional ingestion of cannabis products by children, worsening cognition and psychosis [10], increased exposure to secondhand smoke, and health problems including dependence and respiratory disorders [1,8,11,12] are becoming critically important. However, there is a lack of rigorous, quality data on health outcomes from cannabis use, both alone and when combined with tobacco [13]. Recently, laboratory data have shown that Vitamin E acetate, a component in some Δ9-tetrahydrocannabinol (THC) e-cigarette or vaping products, has been associated with an E-cigarette and Vaping Lung Injury (EVALI) outbreak [14]. Before the growing use of cannabis becomes a substantial public health issue, research is needed to better understand the health impact of its use and to inform state and federal regulatory agencies, health organizations, and health care professionals, so that appropriate action can be taken [1,11].

Only a limited amount of studies examined the concentrations of harmful and potentially harmful constituents in cannabis smoke. Polycyclic aromatic hydrocarbons (PAHs) are generated by the burning of organic matter and are in cannabis smoke [15,16]. PAH concentrations with molecular weight greater than that of chrysene increased significantly in cannabis as compared to tobacco smoke, suggesting an increased cancer risk. In contrast, others reported that PAHs were lower in cannabis smoke [17]. Investigators also reported that nitrogen-containing compounds, including acrylonitrile, ammonia, hydrogen cyanide, NO, NO_x_, and aromatic amines, were higher in cannabis smoke than in tobacco smoke and, in contrast, formaldehyde and acetaldehyde were lower [17,18]. Rickert et al. [19] found that smoking one cannabis joint compared to smoking one manufactured tobacco cigarette yielded higher levels of “tar”, higher pH, and about the same carbon monoxide (CO) under “realistic” smoking conditions: 70 mL puff volume, 5 sec puff duration, and 30 sec puff interval. On a per puff basis, manufactured cigarettes delivered about twice the CO but a similar amount of “tar” as a cannabis joint.

Many studies identified and quantified harmful and potentially harmful constituent (HPHC) biomarker concentrations in cigarette smokers’ urine, including acrylonitrile [20]. However, few investigations evaluated the harmful constituents, besides THC and its metabolites, in blood or urine following cannabis use [21]. Wu et al. [22] concluded that smoking cannabis, regardless of THC content, results in a substantially greater respiratory burden of carbon monoxide and tar than smoking a similar quantity of tobacco. Biomarkers of exposure to cannabis smoke were compared between users and non-users using data from the National Health and Nutrition Examination Survey (NHANES) [23]. In this study, concentrations of many metabolites of individual monohydroxy PAHs, thiocyanate, acrylonitrile, and acrylamide increased in the urine of recent cannabis users compared to non-users. Smith et al. [24] examined biomarkers of exposure to nicotine, tobacco-specific nitrosamines, volatile organic compounds, and PAHs and determined that, among non-tobacco products users, only urinary N-acetyl-S-(2-cyanoethyl)-L-cysteine (a biomarker of acrylonitrile) concentrations were elevated in past-30-day cannabis users compared to non-cannabis users. The International Agency for Research on Cancer (IARC) determined that acrylonitrile is possibly carcinogenic to humans (class 2B) and may present a particular exposure concern for cannabis users because of its possible carcinogenicity and high concentration in cannabis smoke compared to cigarette smoke [19,25]. Breath-holding in cannabis smokers was found to be associated with increased blood carboxyhemoglobin, inhaled and deposited tar, serum THC, and subjective effects in controlled topography exposures [26]. Non-THC-related constituent exposure is important to include in any assessment of possible harm from cannabis use.

Previous studies on non-THC-related exposure were limited in scope and applicability, so that there remains significant uncertainty on the likelihood that these ancillary exposures present a substantial source of risk to users. In the most relevant previous studies described above, investigators examined acrylonitrile urinary metabolites in cannabis users [23,24]. However, the length of time between use and sample collection were nonspecific, asking only whether participants used cannabis that day. Subjects were not asked about recency of last use, frequency of use, or what product they used. Subjects may have been exposed to other sources of acrylonitrile exposure that were not adequately addressed in the exposure questions. The study by Wei et al. [23] used modeling to remove the contribution of cigarette smoking and, while useful, this approach may not have adequately addressed this confounder.

In order to assess the health risk from cannabis use, we need to know the levels of acrylonitrile exposure from a single cannabis use, which routes increase exposure, and whether the acrylonitrile exposure is limited to combustion. Understanding the time dependence of urinary excretion of acrylonitrile exposure biomarkers would inform sample collection and inform interpretation of results [27]. This research examined creatinine-corrected urinary concentrations of N-acetyl-S-(2-cyanoethyl)-L-cysteine (2CYEMA), a major metabolite of acrylonitrile [28], over approximately 48 h following controlled smoked, vaped, and oral cannabis administration of 50 mg THC in occasional and frequent cannabis users.

## 2. Materials and Methods

### 2.1. Study Design

The study was a within-subject, randomized, double-blind, double-dummy, and placebo-controlled investigation, with each participant consuming products that contained 50 mg THC by three different routes of administration, and a placebo. The primary objectives of the study were to evaluate cannabinoid pharmacokinetics and pharmacodynamics following different administration routes in chronic frequent and occasional cannabis users. Detailed descriptions were published previously [29,30] and are summarized below. Additional biomarkers of cannabis exposure were measured as secondary targets and have been reported previously [29].

### 2.2. Subjects

The ten participants in the additional biomarker exposure study were healthy adult volunteers aged 18 to 50 years, who provided informed written consent to participate. The study was approved by Institutional Review Boards of the National Institute on Drug Abuse, Food and Drug Administration, and the Drug Enforcement Administration. Details on the subjects, inclusion and exclusion criteria, and confinement have been provided previously [28]. Subjects were included if they self-reported a mean cannabis intake frequency ≥2 per month but ≤3 per week (occasional users) or ≥5 per week (frequent users) over the previous 3 months and had a positive urine cannabinoid screen (frequent users). Participants entered the secure research unit approximately 19 h before controlled cannabis administration and resided there throughout the study to eliminate exposure to cannabis, but subjects were allowed to smoke tobacco cigarettes during this period.

### 2.3. Exposures and Sample Collection

Cannabis cigarettes, which are rolled like a joint and do not include a filter, were acquired from the National Institute on Drug Abuse Drug Supply Program. Active cannabis cigarettes weighed 0.734 ± 0.05 g and contained 6.9% (0.95%) (approximately 50.6 mg) THC. Placebo cigarettes weighed 0.713 ± 0.05 g and contained 0.001% (0.000%) THC. The Volcano© Medic (Vapormed GmbH and Co., Tuttlingen, Germany) vaporizer was used for vaping of cannabis and operated according to the manufacturer’s specifications. THC vapor was produced using plant material from one 6.9% THC cigarette, ground using an herb mill/grinder provided by the Volcano manufacturer, and heated to a temperature of 210 °C by the hot air generator to vaporize THC. The vapor was collected in a plastic balloon attached to a mouthpiece that allowed the subject to control inhalation. Each session utilized a new balloon to ensure hygienic administration. Cannabis brownies were prepared with the same cannabis material as smoking and vaping. Each brownie contained 50.6 mg THC. Plant material was ground to a fine powder before addition to the Duncan Hines^®^ double-fudge brownie mix, which was then prepared according to the manufacturer’s instructions and baked for 30 min at 121 °C. Each portion contained the equivalent of one cigarette. After baking and cooling, the brownie portions were frozen until use. The portion was removed the night before administration and allowed to thaw in a refrigerator overnight.

During each of the four sessions, one of the three active doses of cannabis or the placebo was administered. The order of administration was random. Subjects were instructed to consume as much as they could from the dose and were encouraged to consume the entire dose within 10 min. Urine samples were collected before and up to 54 h post dose for occasional smokers and up to 72 h post dose for frequent smokers. Participants collected all voided urine ad libitum as individual specimens in polypropylene bottles throughout their stay in the unit. Specimens were refrigerated until checked for total volume, pH, specific gravity, and creatinine. Aliquots were stored frozen at −20 °C until analysis for acrylonitrile biomarkers. Subjects were required to leave the unit for ≥72 h before the next session.

### 2.4. 2CYEMA Analysis

Urinary 2CYEMA was quantified by ultrahigh-performance liquid chromatography coupled with electrospray ionization tandem mass spectrometry with a calibration range of 0.05–150 ng/mL and a detection limit of 0.50 ng/mL, as previously described [31]. To account for differences in urine dilution, values were creatinine-corrected. Urine samples with creatinine concentrations outside the range of 10–500 mg/dL were rejected. This resulted in the rejection of two results. One other result was an evident outlier and rejected; 2CYEMA (18.4 µg/g creatinine, 9 times higher than expected based on data at times immediately before and after, 2.1 and 1.9 µg/g creatinine), THC-glucuronide (detectable whereas points before and after were not detectable), and THCCOOH-glucuronide (3 times higher than expected based on data at times immediately before and after) were all much higher than expected. Urinary cotinine was determined as described previously [32]. The urinary nicotine cutoff for cigarette smoker versus non-smoker was 50 ng/mL [33].

### 2.5. Statistical Analysis

Simple data manipulation and analyses were performed in Microsoft Excel for Office 365. Nonlinear regression analysis of 2CYEMA versus time was accomplished in SAS version 9.4 PROC NLIN using the equation:

2CYEMA (corrected) = a ∗ (exp(−time/c)) + b(1)

where a is the time 0 increase above baseline (µg/g creatinine), b is the baseline (estimated level at infinite time, µg/g creatinine), and c is the half-life (h).

## 3. Results

**Figure 1:**
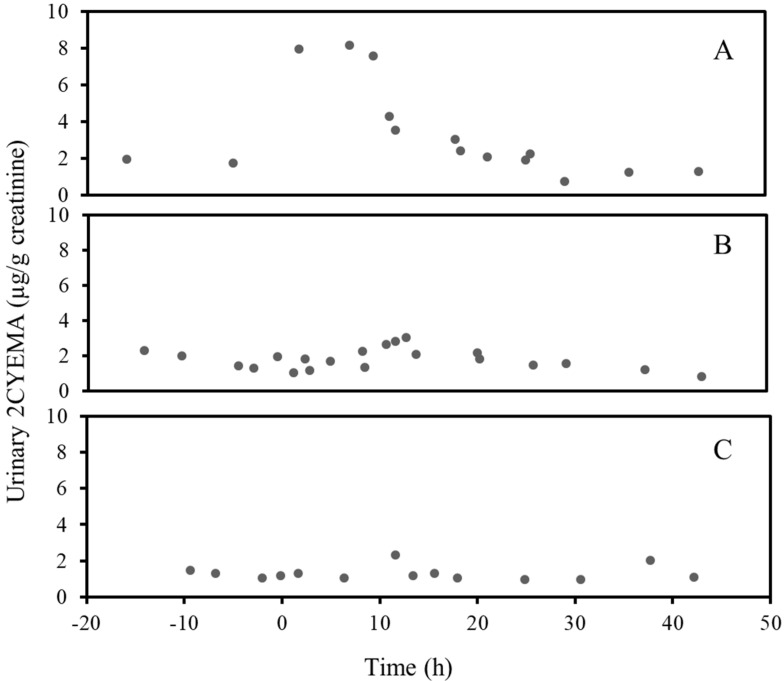
Creatinine-corrected urine 2CYEMA (µg/g creatinine) concentrations over time before and after smoking of a cannabis joint (**A**) vaping of cannabis (**B**) or ingestion of a cannabis brownie (**C**) by an occasional cannabis user.

Figure 1A shows the change in 2CYEMA over time before and after smoking one cannabis joint by an occasional cannabis user. For this subject, 2CYEMA increased immediately after use and reached a maximum concentration of approximately 8.2 µg/g creatinine about 7 h after the start of smoking. Over the next 30 h, 2CYEMA decreased until reaching a baseline of approximately 1.2 µg/g creatinine. Conversely, immediately after this same occasional cannabis user vaped cannabis, 2CYEMA did not increase (Figure 1B). The data show a maximum 2CYEMA concentration at 13 h and a slow decrease in 2CYEMA over the next 30 h. 2CYEMA concentrations over time before and after ingestion of a cannabis brownie by this same occasional cannabis user did not change substantially (Figure 1C).

On two different days, each cannabis user smoked a single cannabis joint or vaped an equivalent amount of cannabis in 10 min. Table 1 provides 2CYEMA for the sample collected at the time immediately before product use, the maximum 2CYEMA achieved following initiation of product use (C_max_), and time to reach maximum 2CYEMA (T_max_) for all 10 subjects. In most cases, the maximum occurred within 4 h of the initiation of cannabis use. In some cases, there was not a rapid increase in 2CYEMA following use or a clear decrease after exposure ended; the maximum value may have occurred later.

Pre-exposure 2CYEMA in occasional cannabis users ranged from 0.84 to 29.2 µg/g creatinine. Following smoking a cannabis joint, these concentrations increased to between 8.2 and 69.0 µg/g creatinine, an increase ranging from 4.1 to 39.8 µg/g creatinine, or a percent increase from pre-exposure to maximum concentration for each subject ranging from 55.4% to 1570%. When these subjects vaped cannabis, the pre-exposure 2CYEMA was similar (1.1–15.5 µg/g creatinine). However, post exposure 2CYEMA concentrations increased only to 1.5–15.6 µg/g creatinine. The differences in 2CYEMA before and after exposure ranged from −0.4 (all post exposure concentrations were lower than the pre-exposure concentrations) to 1.2 µg/g creatinine and the percent increases ranged from −12.5% to 75.0%. For comparison, the pre-exposure concentration was 1.2 µg/g creatinine and the C_max_ post exposure concentration was 2.3 µg/g creatinine with a T_max_ of 11.6 h for subject K when consuming the THC-containing brownie.

For frequent cannabis users, the pre-exposure 2CYEMA before smoking a joint was between 23.3 and 204 µg/g creatinine and showed a wide range of differences post exposure (−38.0 to 86.1 µg/g creatinine, −35.6% to 149%). For the two subjects with the highest pre-exposure 2CYEMA, no post exposure 2CYEMA was greater than the pre-exposure level. The difference between the post exposure maximum 2CYEMA and the pre-exposure 2CYEMA for frequent cannabis users following cannabis vaping ranged from −26 to 1.5 µg/g creatinine (−36.0% to 6.4%). No substantial increases in 2CYEMA were seen in any of these five subjects under these conditions.

Urinary cotinine differentiated cigarette smokers and non-smokers among the ten subjects. All frequent cannabis users were also cigarette smokers. Pre-exposure 2CYEMA in the four non-cigarette smokers ranged from 0.84 to 7.4 µg/g creatinine. Pre-exposure 2CYEMA in the six cigarette smokers ranged from 23.3 to 204 µg/g creatinine. Pre-exposure 2CYEMA was higher in the cigarette smokers, including the one occasional cannabis user, than the range of 2CYEMA in non-cigarette smokers, despite the 19 h cannabis washout period; participants were permitted to smoke cigarettes during the non-protocol pre-exposure period, which likely contributed to variation in the initial 2CYEMA.

**Figure 2:**
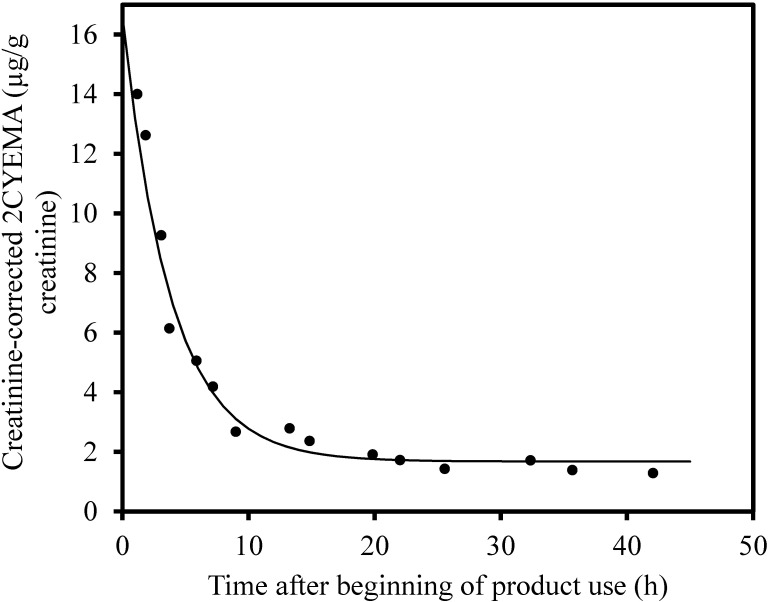
Post exposure creatinine-corrected urine 2CYEMA (µg/g creatinine) over time following smoking of a cannabis joint by an occasional cannabis user with a best-fit exponential curve.

Figure 2 shows a nonlinear exponential fit of 2CYEMA versus time after the beginning of smoking cannabis for subject X. The data are well characterized by the nonlinear curve and yield a 2CYEMA increase above baseline at time 0 of 14.9 µg/g creatinine, a baseline of 1.7 µg/g creatinine, and a half-life of 3.9 h. Uncertainty in the data does not preclude the possibility of more complex excretion kinetics.

Table 2 shows the results of the nonlinear exponential fit of 2CYEMA versus time after the beginning of exposure for both occasional and frequent cannabis users. The data for subject D failed to converge and the nonlinear exponential fits for subject L and T were not statistically significant. In agreement with the data provided in Table 1, both baseline and 2CYEMA at time 0 above baseline for non-tobacco smoking occasional cannabis users were lower than the range of frequent users and/or cigarette smokers.

## 4. Discussion

These results demonstrate that urinary 2CYEMA increases rapidly in non-smoking occasional cannabis users. The quantitative increase in 2CYEMA in this subset of participants was fairly consistent, ranging from 4.1 to 20.2 µg/g creatinine. Because users were free to smoke cannabis as intensely as they chose in the 10 min period, it is likely that the range of 2CYEMA increases results from differences in smoking topography. Peak nicotine concentrations in users of e-cigarettes vary considerably due to smoking behavior [34,35] and similar factors are likely affecting the 2CYEMA results in this study. Wei et al. [23] previously reported the geometric means of 2CYEMA in the urine of non-users of tobacco (1.44 (95%CI: 1.34, 1.56) ng/mL), cannabis users (15.4 (95%CI: 8.15, 29.0) ng/mL), and cigarette smokers (24.0 (95%CI: 15.0, 38.5) ng/mL). If one assumes an average creatinine concentration of 100 mg/dL for the results from Wei’s study [24], our pre-exposure results for non-smoking occasional cannabis users were generally between their reported biomarker concentrations for 2CYEMA in non-users of either tobacco or cannabis and cannabis users. Post exposure maximums for our non-smoking occasional cannabis users are within their reported range of 2CYEMA in cannabis users [23]. Post exposure maximum 2CYEMA for frequent cannabis users (who were also tobacco cigarette smokers) are similar or higher than their results. This is as expected since, in our study, these subjects were all dual cannabis/cigarette users and samples were collected immediately after cannabis use.

Vaping cannabis did not consistently increase 2CYEMA across users and the differences between pre- and post exposure 2CYEMA were small and unpredictable. For several of the subjects, including one occasional user, 2CYEMA immediately before vaping was higher than any 2CYEMA results following use. This suggests that, in contrast to cannabis joint smoking, vaping cannabis does not lead to substantial exposure to emissions of acrylonitrile from a single bout. Since vaping heats cannabis at a lower temperature than smoking either cannabis or tobacco cigarettes, acrylonitrile may not be generated or volatilized and released into the inhaled aerosol at this lower temperature. While there are numerous harmful and potentially harmful constituents (HPHCs) generated in cannabis smoke, higher concentrations of acrylonitrile in combusted cannabis smoke compared to non-combusted cannabis aerosol may suggest an additional cancer risk, even though its harm relative to other HPHCs is undetermined at this time.

Smoking cannabis joints significantly increased 2CYEMA in the urine of occasional cannabis users. However, in frequent cannabis users, the difference between post and pre-exposure 2CYEMA was not consistently positive. This is likely due to confounding from tobacco smoking during the pre-protocol period, since all frequent cannabis users were also tobacco cigarette smokers. The three frequent cannabis use participants with lower pre-exposure 2CYEMA did show a measurable increase following cannabis smoking. However, for the two participants with higher pre-exposure 2CYEMA, the pre-exposure concentration was higher than any of the post exposure concentrations. This may also be related to differences in product use behavior, since more intense smoking should lead to higher biomarkers of exposure. However, analysis of the relationship between THCCOOH-glucuronide and 2CYEMA post exposure for all subjects did not show a statistically significant association as would have been expected if intensity of use were a dominant factor.

For the seven subjects with 2CYEMA data after cannabis smoking that could be successfully fit to a nonlinear exponential, the half-lives ranged from 2.5 to 9.0 h. Jakubowski et al. [36] examined 2CYEMA excretion kinetics in six participants following 8 h of acrylonitrile exposure by inhalation of gas generated in an exposure chamber for up to 31 h after exposure with no known other sources of exposure. In their investigation, elimination approximated first-order kinetics and had a mean half-life of approximately 8 h, within the range determined in our study. The higher mean half-life determined by Jakubowski in comparison to much of our data may have been due to the different times of exposure and the previously unexposed nature of the study subjects. Increasing exposure time in their study may have allowed deposition into deeper body stores that would require a longer time for elimination.

Goniewicz et al. [27] examined the half-life of 4-(methylnitrosamino)-1(-3-pyridyl)-1-butanol (NNAL), the metabolite of a known carcinogen in tobacco smoke, in the urine of eight daily (>15 per day) and five occasional tobacco smokers (<20 per month). They found that the non-compartmental half-life for daily cigarette smokers was 9.1 days and for occasional cigarette smokers, 16.0 days. The investigators suggested the difference between occasional and frequent cigarette smokers was deposition in deep tissue compartments with a long terminal half-life. Although the half-lives were different for the two chemicals studied, we found a similar result in our data, since the half-lives of 2CYEMA in frequent cannabis users/cigarette smokers ranged from 2.5 to 3.1 h whereas the half-lives of 2CYEMA in occasional cannabis users ranged from 3.8 to 9.0 h. The data in Figure 2 suggest that the elimination kinetics may be multiexponential, but the data collected here were not adequate to make this determination for most of the participants. Boogaard and van Sittert [37] reported that the half-life of S-phenyl mercapturic acid, a metabolite of the tobacco smoke-related volatile organic compound, benzene, following inhalation was 9.1 ± 0.7 (SE) h, which is in line with our findings. Fuhr et al. [38] examined the elimination kinetics of mercapturic metabolites of acrylamide after ingestion. The half-life of N-acetyl-S-(2-carbamoylethyl) cysteine was 17.4 ± 3.9 (SD) h and for N-acetyl-S-(2-hydroxy-2-carbamoylethyl) cysteine, 25.1 ± 6.4 (SD) h. However, elimination following ingestion would be longer due to the time required for absorption in the gastrointestinal tract.

The study was strengthened by the confinement of subjects and use of cannabis in a controlled setting. The collection of all urine samples up to 54–72 h post dose (at least six half-lives) allowed for better determination of the baseline 2CYEMA, which helps in modeling elimination kinetics and half-life determination. The use of products with the same amount of THC and over the same time period allowed a direct comparison between routes of exposure and between subjects.

However, there were limitations to this study. The urine elimination of 2CYEMA was examined in only ten participants in this study, limiting the generalizability of the findings. While this study evaluated three different routes of consumption, it examined only a single amount of cannabis, 50 mg. Other levels, either higher or lower, could lead to different results, particularly relative to the amount of acrylonitrile to which cigarette smokers are exposed. The use behavior of the products was only restricted by a maximum amount of time, during which subjects were required to consume the products, but this could also be considered a strength since this was natural instead of controlled use. If participants smoked the entire joint in 4 min, the rate of increase and maximum 2CYEMA attained may differ substantially from a subject who used the entire 10 min period to consume the product. Most of the subjects were tobacco smokers and acrylonitrile is present in cigarette smoke. Because all frequent cannabis users were also cigarette smokers, we cannot differentiate these two sources of confounding. The pre-exposure levels of 2CYEMA in many cases were not low, even after 19 h of THC washout, likely because participants were allowed to smoke tobacco during the pre-exposure period. High initial 2CYEMA may have obscured increases in 2CYEMA in participants who smoked tobacco when vaping cannabis.

## 5. Conclusions

By collecting and analyzing 2CYEMA in samples collected before and after smoking, vaping, or eating cannabis, the study showed that smoking a cannabis joint markedly increases 2CYEMA within 10 min in occasional users. Vaping or eating cannabis does not appear to alter 2CYEMA levels substantially. However, the boiling point of acrylonitrile is 77.3 °C, so the acrylonitrile may have no longer been present when consumed in brownies. Therefore, acrylonitrile either must be a combustion product of cannabis use or released from the cannabis during the elevated temperatures encountered during combustion. The half-lives of 2CYEMA range from 2.5 to 9 h and vary depending on previous exposure to acrylonitrile, perhaps through frequent smoking of tobacco or cannabis use. In order to interpret exposure accurately in cannabis smokers using 2CYEMA, details about tobacco smoking and the time since last use of cannabis should be collected and included as a covariate in any subsequent analysis.

## Figures and Tables

**Table 1:** Creatinine-corrected urinary 2CYEMA concentrations in occasional and frequent cannabis users immediately before product use, maximum concentrations (C_max_), and time to maximum concentration (T_max_) following product use.

Subject	Smoked Cannabis				Vaped Cannabis			
	Pre-Exposure	C_max_ Post Exposure (µg/g Creatinine)	% Difference	T_max_ (h)	Pre-Exposure	C_max_ Post Exposure (µg/g Creatinine)	% Difference	T_max_ (h)
	Occasional Users							
K	1.7	8.2	382	7.1	2.0	3.2	60.0	12.8
P *	29.2	69.0	136	2.2	15.5	15.6	0.6	0.7
U	7.4	11.5	55.4	2.4	1.1	1.5	36.4	9.8
W	3.5	23.7	577	3.8	3.2	2.8	−12.5	5.0
X	0.84	14.0	1570	1.2	1.2	2.1	75.0	3.9
	Frequent Users							
D *	23.3	30.0	28.8	3.8	34.2	34.7	1.5	2.4
G *	65.2	42.0	−35.6	6.6	44.5	28.5	−36.0	20.0
L *	25.9	52.0	101	3.8	26.6	24.3	−8.6	1.2
T *	204	166	−18.6	0.4	140	114	−18.6	0.6
Y *	57.9	144	148	1.3	23.4	24.9	6.4	3.1

* Cigarette Smoker.

**Table 2:** Results of the nonlinear exponential fit of creatinine-corrected urinary 2CYEMA concentrations (µg/g creatinine) versus time after the beginning of product use.

Occasional Users	Time 0 Increase above Baseline (µg/g Creatinine)	Baseline (µg/g Creatinine)	Half-Life (h)	Pr > F
K	23.2	1.4	6.0	0.0005
P *	101	19.8	3.1	<0.0001
U	13.0	2.3	9.0	0.0001
W	32.9	3.1	7.4	<0.0001
X	14.9	1.7	3.8	<0.0001
Frequent Users				
D *	Did not converge **			
G *	163	25.0	3.9	0.020
L *	33.0	26.8	10.1	0.14
T *	87.4	93.0	2.5	0.30
Y *	309	33.4	2.5	0.0007

* Cigarette Smoker. ** Nonlinear regression analysis could not reach a minimum sum of squared residuals for the model.
